# A collaborative care package for depression comorbid with chronic physical conditions in South Africa

**DOI:** 10.1186/s12913-022-08874-7

**Published:** 2022-12-01

**Authors:** Inge Petersen, One Selohilwe, Daniella Georgeu-Pepper, Christy-Joy Ras, Babalwa Zani, Ruwayda Petrus, Lauren Anderson, Ntokozo Mntambo, Tasneem Kathree, Arvin Bhana, Graham Thornicroft, Lara Fairall

**Affiliations:** 1 Centre for Rural Health, School of Nursing and Public Health, University of KwaZulu-Natal, Howard College, Mazisi Kunene Road, Durban, 4001 South Africa; 2 Institute for Global Health, University College London, London, UK; 3 Knowledge Translation Unit, University of Cape Town, Cape Town, South Africa; 4 School of Applied Human Sciences, University of KwaZulu-Natal, Howard College, Mazisi Kunene Road, Durban, 4001 South Africa; 5 Health Systems Research Unit, South African Medical Research Council, 491 Peter Mokaba Ridge, Overport, Durban, South Africa; 6 Centre for Global Mental Health and Centre for Implementation Science, Institute of Psychiatry, Psychology and Neuroscience, King’s College, London, UK; 7 Global Health Institute, King’s College London, London, UK

**Keywords:** Task-sharing, Co-designed collaborative care model, Integrated mental health care, Pragmatic cluster randomized controlled trial

## Abstract

**Introduction:**

A task-sharing collaborative care model for integrated depression care for South Africa’s burgeoning primary health care population with chronic conditions was developed and tested through two pragmatic cluster randomized controlled trials. One trial focused on patients with hypertension and was located in one district where a collaborative care model was co-designed with district stakeholders. The other trial, focused on patients on antiretroviral treatment, was located in the same district site, with the addition of a second neighbouring district, without adaptation of the original model. This paper describes the package used to implement this model, and implementation outcomes across the two sites, and summarises lessons and challenges.

**Methods:**

The Template for Intervention Description and Replication (TIDieR) framework, adapted for complex health systems interventions, was used to describe components of the package. Additional elements of ‘modifications made’ and ‘actual implementation’ introduced in the ‘Getting messier with TIDieR’ framework, were used to describe implementation outcomes in terms of reach, adoption and implementation across the two trial districts.

**Results:**

In the absence of a co-design process to adapt the model to the context of the second site, there was less system level support for the model. Consequently, more project employed human resources were deployed to support training of primary care nurses in identification and referral of patients with depression; and supervise co-located lay counsellors. Referrals to co-located lay counselling services were more than double in the second site. However, uptake of counselling sessions was greater in the first site. This was attributed to greater in-vivo supervision and support from existing mental health specialists in the system. There was greater reliance on online supervision and support in the second site where geographical distances between clinics were larger.

**Conclusion:**

The need for in-country co-designed collaborative care models, and ‘implementation heavy’ implementation research to understand adaptations required to accommodate varying in-country health system contexts is highlighted.

**Supplementary Information:**

The online version contains supplementary material available at 10.1186/s12913-022-08874-7.

## Background

South Africa and many other low-and-middle-income countries (LMICs) face an epidemiological shift in the disease burden towards chronic conditions [1]. South Africa has high rates of HIV, hypertension, diabetes, and common mental disorders (depression, anxiety, substance use disorders) (CMDs). Prevalence estimates for HIV in the 15–49 year age group was 19,6% in 2022 [2]; for hypertension and diabetes, it was 37.1% and 11.4% respectively in the ≥ 15 year population in 2016, with underdiagnosis estimated to be close to 50% for hypertension and 61% for diabetes [3]. Added to this, one in three people experience CMDs during their lifetime [4], with a treatment gap of 92% in public sector outpatient services [5]. Depression is the most prevalent single condition, with a lifetime prevalence of 9.7% [6]. HIV and tuberculosis (TB) often co-exist with non-communicable diseases (NCDs), especially cardiovascular disease, diabetes, and CMDs [4, 7–9]. Estimates of depressive symptoms in people living with HIV on antiretroviral treatment in sub-Saharan Africa (SSA) varies from between 14 and 32%, depending on the measure used [10]. Comorbidity of NCDs in people with depression is common, with a study in the Western Cape finding that 80% of people with depression had at least one of the following NCDs: hypertension, diabetes, respiratory disease [9]. While co-occurring conditions are fewer in younger age groups, one in five South Africans have co-occuring conditions, with females and older adults being most affected, but also observed in younger adults and attributed to the high prevalence of HIV and hypertension [11].

Mental health conditions comorbid with physical conditions, and depression in particular, result in increased risk for poor physical health outcomes and increased health care costs compared to chronic care patients without associated CMDs [12–14]. The integration of mental health care into chronic service delivery platforms in primary health care (PHC), and in particular for depression, is thus critical to improving overall health outcomes and containing the cost of chronic care in LMICs, particularly for people living with HIV as well as the growing number with NCDs.

In response to the growing multimorbidity problem in South Africa, the National Department of Health (NDoH) introduced Integrated Clinical Services Management as as part of re-engineering of primary health care (PHC) [15]. Integrated Clinical Services Management is a health system strengthening model that builds on the strengths of the South African HIV programme to deliver integrated primary care to patients with any chronic disease [16]. It includes training of professional nurses, who provide 90% of public sector primary healthcare in South Africa [17], in the content of the World Health Organization’s mental health Gap Action Programme (mhGAP) guidelines. These are part of an integrated clinical decision support tool called Adult Primary Care (APC), for the management of adults presenting with NCDs, communicable diseases, mental illness and women’s health [18]. However, previous research has shown that in the case of depression this training should be accompanied by system strengthening interventions to improve case detection [19].

Given the evidence from high- and middle-income countries of the cost-effectiveness of collaborative stepped care for co-existing mental physical conditions in PHC [20–22], a system strengthening collaborative package for chronic patients with co-existing depression was co-developed, and pilot tested in preparation for evaluation through two parallel hybrid type 1 randomized control trials. One of the trials, the Co-morbid Affective HIV Long-Term health (CobALT) trial focused on the effect of this package on depression and viral load suppression in people on antiretroviral treatment (ART); and the other, the Programme for Improving Mental Health Care – South Africa (PRIME-SA) trial, on depression outcomes and hypertension control in people on treatment for hypertension [23, 24]. The CobALT trial was located in 20 intervention and 20 control facilities across two neighbouring district sites. The PRIME-SA trial was located in the 10 intervention and 10 control facilities in one of the two district sites, where the formative work informing the development of the collaborative care model and associated implementation package was conducted.

The aim of this manuscript is to describe the intervention tested in the trials and better understand how context impacted on implementation of the package and service delivery outcomes across the two district sites. This is in line with type 1 hybrid trials [25] with the view to first, induce implementation lessons and challenges that may assist the interpretation of the trial findings. Second, to assist in narrowing the research-to-practice gap should the implementation package be introduced in other contexts or taken to scale as suggested by Rudd et al. [26].

## Methods

### Setting

The Dr Kenneth Kaunda (Dr KK) district in the North West province of South Africa, formed the site for the PRIME-SA trial as well as the first site for the phased CobALT trial, with the the second site being the province’s neighbouring Bojanala Platinum (Bojanala) district. Dr KK was a pilot district site for National Health Insurance (NHI) and as a consequence garnered additional resources such as NHI PHC doctors [27]. Bojanala was selected as the second site for the CobALT trial given that it conveniently bordered on Dr KK, was geographically larger, with almost twice the population of Dr KK and a large HIV population, with the CobALT trial powered to recruit a larger sample than the PRIME trial. Additional details of the sites can be found in Table 1.

**Table 1:** Description of Dr Kenneth Kaunda and Bojanala Platinum district

		Dr Kenneth Kaunda District	Bojanala Platinum District
Population		742 821	1 657 148
Unemployment rate		29.7%	25.6%
Poverty headcount		4.9%	8.8%
NHI pilot site		Yes	No
ICSM pilot site		Yes	No
Number of clinics		60	115
Leading causes of mortality	HIV & TB burden	28%	22%
	NCDs	47%	47%

Sources: http://cs2016.statssa.gov.za/wp-content/uploads/2018/07/NorthWest.pdf; http://www.nwpg.gov.za/VTSDEconomy/Documents/VTSD%20Profile/Profile%20Bojanala%20District.pdf; https://www.hst.org.za/publications/NonHST%20Publications/North%20/West%20-%20KK%20Kaunda%20District.pdf

### Approach

The World Health Organization (WHO) continuum of implementation research [28] frames the development, evaluation and implementation of the PRIME/CobALT mental health integration package into routine practice, with the two trials located in the middle of this continuum. Between 2011 and 2014, there was an intensive formative phase in one of the two districts (Dr KK), with the PRIME trial restricted to this district. This formative work included a situational analysis; and qualitative research with providers, patients and managers, culminating in theory of change workshops, where a collaborative care model was co-designed with key provincial and district stakeholders [29], integrated into a district mental health plan and then pilot tested [30]. This formative work was not replicated in the Bojanala district due to resource and time constraints. The purpose of the two trials, was to test the effectiveness of the intervention as part of routine practice.

### Control condition

The control condition in both trials involved the Integrated Clinical Services Management model described in the introduction and implemented by the Department of Health prior to the trials in both sites. Professional PHC nurses were, at the time of the trials, not authorized to prescribe psychotropic medication in South Africa. Consequently, in the control condition, they used APC to provide psychoeducation and referred patients they identified as having depression to stationed/ambulatory PHC doctors servicing PHC facilities (the norm in South Africa being 4 h per week at the time of the trials^1^ for the initiation of antidepressants; and/or to limited specialist mental health services, mostly at district hospital level, for more specialist mental health treatment. This was in addition to their role of managing psychiatric emergencies requiring acute sedation and/or hospital admission.

Implementation of the model included reorganization of patient flow to include a dedicated stream for patients with chronic conditions and training of Professional PHC nurses in APC (described in the introduction). Historically, the 4-year pre-service training of professional nurses in South Africa prepares them to work in hospitals alongside doctors, but not as independent clinicians in primary care. APC guidelines and accompanying in-service training aims to equip professional nurses to work as independent PHC practitioners in PHC settings. The guide comprises a concise set of algorithms and checklists that cover all common symptoms and chronic conditions in adults presenting to primary care, and is updated every two years [19]. Training in APC followed a cascade and phased approach whereby facility trainers selected from clinics were trained by Master Trainers drawn from manager and educator pools at provincial, district and sub-district level; who were in turn, trained and mentored by Provincial Regional Training Centres (RTCs). APC training, described in detail elsewhere [31], comprised 27 structured case scenarios delivered through 12 (1.5–2 h), onsite, interactive training sessions that integrated content on NCDs, communicable diseases, mental illness and women’s health (two of the training sessions were on mental illness covered by three cases, including depression), that were worked through using the APC guide available at the time.

### Intervention condition

The intervention tested in the trials was scaffolded off this control condition in both districts using a system strengthening approach [28] and existing personnel wherever possible. The intervention was located at district and facility levels, with the specific goals to strengthen the *capacity* of the PHC workforce for integrated primary mental health care; and to strengthen within-clinic *referral pathways* to include co-located counselling services provided by lay-counsellors. The latter was introduced given: i) a paucity of mental health specialists available to provide psychological counselling interventions at PHC level, with ample evidence of the effectiveness of task-shared lay-counselling services in LMICs [32]; as well as ii) limited availability of PHC doctors to initiate psychotropic medication in PHC settings, with initiation beyond the scope of practice of professional nurses in South Africa at the time of the study.

We used the Template for Intervention Description and Replication (TIDieR) framework for complex health systems interventions [33], to guide the description of the resulting intervention and implementation package to aid future replication [33].

The ‘Getting Messier with TiDIER’ framework has been found to be a useful tool for capturing changes and adaptations that occur over the course of projects in response to contextual changes [34]. We used the additional element of ‘actual implementation’ to identify actual implementation of the package and ‘modifications made’ across the two sites together with the RE-AIM framework [35]. Notably, we focused on the reach and adoption of the training provided to the various providers in the system; as well as reach and adoption of the package in relation to patients as part of routine services. ‘Modifications made’ enabled us to report on the implementation element of the RE-AIM framework in relation to adaptations made across the two sites.

We describe below the intervention for each service provider within the system as well as the implementation strategies to implement the intervention.

### Professional nurses

#### Intervention

Within the collaborative care model that was tested in the two trials, nurse capacity to screen, diagnose and refer patients with depression was strengthened. Referral pathways for treatment initiation by PHC doctors or specialist mental health services remained the same as in the control condition. However, in the intervention condition, this was augmented by including a referral pathway to lay-counsellors for psychological counselling. Further, they were also capacitated to provide a case management function, reviewing patient progress and referring complex and treatment resistant cases to specialist mental health services.

#### Implementation strategies

In the intervention trial clinics, the standard APC training was strengthened by a supplementary APC Mental Health Module to better equip nurses to use APC to screen for and assess, advise and refer appropriately any patient with a common mental health condition. The Mental Health module comprised four additional onsite sessions on mental healthcare, using nine mental health case scenarios. These scenarios focused on patients with various conditions designed to increase the users’ confidence in diagnosing mild depression; diagnosing and treating moderate-severe depression; diagnosing and treating depression in a patient with other chronic conditions, including HIV; dealing with non-adherence to treatment due to depression; managing a suicidal patient; diagnosing and managing substance abuse; and managing a depressed patient who was not responsive to treatment. The training also highlighted the distinction and relationships between social stressors, especially poverty, and depression, as pilot work had showed that nurses tended to prioritise referral to social welfare ahead of providing clinical care for people presenting with depression [29]. In addition, it addressed the stigmatised nature of mental illness, providing a non-judgemental space in which to speak candidly about working, and living with, people with mental illness. In relation to case management, the guidelines required nurses to review patients with depression after 8 weeks to assess response to treatment and onward referral for specialist care following a treatment-to-target approach, as contained in the collaborative care model. Treatment to target involves tracking a patient’s symptom severity and adjusting or intensifying treatment should patients not show an improvement in symptoms following initial treatment. The format of the training followed the same cascade model of training used for basic APC training. District Master Trainers trained Facility Trainers to equip them to deliver the four onsite sessions to their co-workers at facilities. (see online supplement using TIDieR framework for additional details).

In response to initial formative work, where the need for non-technical skills was identified to orientate and equip nurses with skills for person-centred care [29], the APC Supplementary Mental Health Module was augmented by four sessions on Clinical Communications Skills. These sessions orientated clinic staff to the collaborative care model and the Department of Health Integrated Clinical Service Management model. They also provided training in clinical communication skills necessary for person-centred care that takes into consideration the emotional and physical well-being of patients, providing PHC nurses with skills to cope with emotional labour, as well as capacitating them in motivational interviewing skills to encourage patient self-management. The sessions were run on-site by clinical communication specialists in both districts. (See TIDieR online Supplement 1 for additional details).

### PHC doctors

#### Intervention

While the responsibilities of the doctors in the system remained the same as in the control condition, their capacity to diagnose and initiate antidepressant medication in patients referred to them by Professional Nurses was enhanced in the intervention condition.

#### Implementation strategy

Two-day workshops were held in both districts to i) Orientate PHC doctors to the mental health content of the APC Guide, the collaborative care model, and to the importance of their role in initiating psychotropic medication to PHC patients referred to them; and ii) To strengthen their capacity to manage mental disorders, including initiation and monitoring of antidepressant and psychotropic medication. (See TIDieR online supplement 3 for additional details).

### Lay-counsellors

#### Intervention

Within the intervention condition, lay-counsellors were introduced into the facilities and were responsible for providing a manualized counselling intervention to patients with depressive symptoms referred to them. Based on formative interviews with chronic care patients, the counselling intervention was adapted from a previous lay counselling intervention developed for depression counselling in HIV-positive patients specifically [36]. Formative work identified poverty; interpersonal conflict; social isolation/ avoidance; grief and bereavement and internalised and externalised stigma as common triggers of depression [37]. The intervention was made up of eight sessions and an additional adherence session called “Getting to know your chronic condition(s) and medication”. The initial session provided psychoeducation on depressive symptoms; with the middle sessions (*n* = 6), introducing common triggers of depression in the target community [38] using narrative vignettes; with the last session on closure. Depending on the trigger, evidence-based cognitive behavioural techniques, including problem solving and behavioural activation [39] were used to work through issues raised in the vignettes. A waiting room educational talk delivered by the lay-counsellors, was also introduced to encourage patients waiting for consultations to disclose their symptoms during consultations and make them aware of available depression services in their facility. (see TIDieR online Supplement 3 for additional details).

While existing clinic-based HIV-counsellors were initially identified to provide the co-located counselling for patients during the formative phase [29], after initial testing in Dr KK as part of the formative work, it was determined that this was not feasible as it potentially infringed on the services of pre- and post-test HIV counselling at a time when the UNAIDS 90–90-90 targets were first introduced, and in the context of clear ringfencing of conditional grant funding for HIV programmes [29]. To allow the trial to proceed, lay-counsellors were project-employed.

#### Implementation strategy

Training and Supervision adopted an apprenticeship model involving a week-long off-site training, on-site peer to peer mentoring, in-vivo/remote supervision by a mental health professional (a project-employed psychologist /registered psychological counsellor^2^ or Department of Health psychologists completing their community service). A fidelity checklist adapted from the ENhancing Assessment of Common Therapeutic factors (ENACT) [40] rating scale was initially used to promote competency, with supervisor and lay-counsellor independently providing scores and consensus reached upon discussion. This tool was also used to assess fidelity. Weekly group supervision was provided face-to-face or remotely, where challenging cases were discussed. Further, debriefing/emotional support sessions by psychologists were also provided on a monthly basis to lay counsellors either face-to-face or remotely (see online TIDieR Supplement 3 for additional details).

### District and PHC facility managers

#### Intervention

To enlist the support of district and facility managers in the introduction of the collaborative care model in the intervention facilities, district and facility operational managers were orientated to the collaborative care model and the roles and functions of the different health care providers in the model.

#### Implementation strategy

Half-day orientation workshops were held with managers on implementation of the model, including the on-site training of nurses in the supplementary APC Mental Health material as well as integration of the lay-counselling service. (See online TIDieR Supplement 4 for more details). Table 2 provides an overview of the intervention components and implementation strategies described.

**Table 2:** Overview of intervention components and implementation strategies

Provider	Intervention	Implementation Strategies
Intervention and Control Condition		
Professional PHC nurses	Capacitated to use the Department of Health Basic APC guidelines to identify, provide brief psycho-education and refer patients with depression	**Dr KK and Bojanala** Use of Department of Health Basic APC guidelines Department of Health (DoH) cascade model of training was used where APC Master Trainers provide onsite training in Basic APC guidelines using case scenarios (n = 27) over 12 weekly sessions to capacitate Professional PHC nurses to use the APC guidelines to identify and manage common chronic diseases, including communicable diseases, NCDs, women’s health and mental health (3 of the 27 cases over 2 of the 12 sessions)**.** Mental health components draw on the WHO’s mhGAP guidelines and adopt a syndromic approach to mental health symptoms (such as stress, insomnia, suicidal thinking) with diagnostic algorithms and treatment checklists for depression. A cascade model of training was used where district APC Master Trainers train Facility Trainers who train PHC nurses at the facilities
Intervention Condition only		
Professional PHC nurses	Strengthened capacity to use Department of Health Basic APC guidelines to identify, provide brief psycho-education, refer to facility-based lay counsellors in addition to existing referral pathways and provide case management	**Dr KK and Bojanala** The DoH cascade model of training was emulated where APC Master Trainers trained Facility Trainers who trained Professional PHC nurses to use additional case scenario material of chronic patients with comorbid mental health conditions at the facilities. The scenarios covered the following: i) Detection of depression and anxiety, psychoeducation and referral to lay-counsellors and/or doctors for consideration of psychotropic medication in the case of moderate to severe depression ii) Detection of risky alcohol use and brief intervention for harmful/hazardous drinking. Detoxification and referral to specialist rehabilitation programmes for dependency as per the mhGAP guidelines^1^ iii) Assessment of suicide intent iv) Patient review after 8 weeks of lay counselling services to assess response to treatment and onward referral for specialist care if necessary following a treatment-to-target approach as contained in the collaborative care model. Treatment to target involves tracking a patient’s symptom severity and adjusting or intensifying treatment should patients not show an improvement in symptoms following initial treatment **Bojanala only** Additional project support was provided for facility-based training. The use of the counsellor referral forms were also integrated into the APC training in Bojanala
Professional PHC nurses	Orientation to Integrated Clinical Services Management model & Clinical Communication skills training	**Dr KK and Bojanala** Clinical Communication Skills Workshops Four 2-h interactive workshops at PHC facilities/regional training centres facilitated by Clinical Communication Skills experts covering the following: i) Overview of the system changes being made by the DoH in South Africa to accommodate the demands of integrated chronic care; their role as case managers within the collaborative care model for depression; ii) Orientation to person-centred care and clinical communication skills necessary to implement person-centred care; iii) Skills to manage patient emotions within the consultation; self-care including how to cope with their own emotions and burn-out; iv) Motivational interviewing skills to promote patient self-management **Bojanala only** APC clinical training included signposting the different clinical communication skills
PHC doctors	Strengthened capacity to diagnose, initiate and monitor response to psychotropic medication	**Dr KK and Bojanala** Two-day face-to-face workshops facilitated by psychiatrists and physicians were held to: i) Orientate PHC doctors to the importance of treating comorbid depression ii) Upskill PHC doctors in the use of APC in managing depression and anxiety using case scenarios **Dr KK only** Training in mhGAP guidelines for other conditions besides depression and anxiety
Lay counsellors	Introduced into facilities and capacitated to provide manualized counselling for patients with depression, drawing on problem solving and cognitive behavioural techniques to address the common triggers of depression and anxiety. An additional adherence counselling session.	**Dr KK and Bojanala** One week of off-site training in the use of a manualized 8-session counselling intervention for depression with an additional adherence counselling session facilitated by the project employed clinical psychologist and psychological counsellors with a Bachelor in Psychology using adult education principles; one week of peer to peer mentoring of lay-counsellors in the clinics in the use of the manual; supervision by a psychologist/B.Psych counsellor assisted by a bespoke fidelity checklist of each session—either in-vivo or of recorded sessions; weekly follow up group supervisory sessions (face to face or online), augmented by ad hoc individual supervision/mentoring sessions The training was organized accordingly to mirror the counsellors’ activities when delivering the intervention using the intervention materials and covered the following sections: i. A psychoeducation session on depression offered to all patients referred to the lay counsellor during their first meeting which took place on the day of the referral ii. Using the step-by-step intervention manual to address the identified triggers and issues that maintain depressive cycles (poverty; interpersonal conflict; social isolation/ avoidance; grief and bereavement; internalized and externalized stigma) iii. The closure session iv. Facilitating adherence counselling and providing accurate information on chronic conditions and patients’ prescribed treatment where needed through the use chronic conditions educational pamphlets
District and facility operational managers	Support implementation of collaborative care model in facilities and district	**Dr KK and Bojanala** Once-off half day workshops facilitated by project employed intervention coordinators to orientate district and PHC facility managers to the PRIME/CobALT collaborative model of care and the task-shared counselling intervention **Bojanala only** Individual orientation for each facility manager

### Implementation strategy for the intervention as a whole

**Fig. 1:**
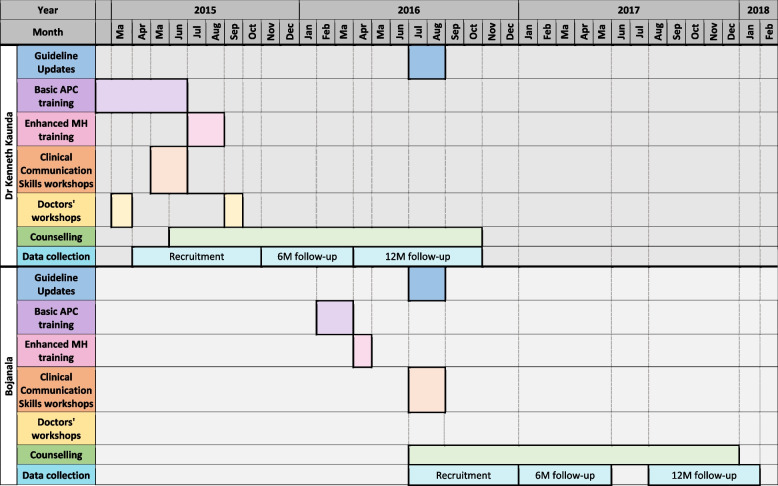
Timeline of intervention implementation activities during the trials

The intervention as a whole was implemented in a phased manner across both sites so as to allow an embedding period before recruitment and enrolment into the trial. Nurse training preceded that of the counsellor training, with training of doctors occurring once the trials had commenced. See timeline in Fig. 1.

### Data collection

Project training records and facility training records were used to assess training coverage, including cascaded APC trainings provided by facility trainers. Project supervision records were used to assess supervision coverage, particularly of the counselling supervision. Counselling intervention fidelity was assessed using fidelity checklists adapted from the ENACT rating scale; and patient counselling uptake assessed from patient tracking forms.

### Results

#### Implementation of orientation of district and PHC facility managers

There were variations in implementation of the orientation package between the Dr KK and Bojanala. In Dr KK, a half-day workshop was held with all district and intervention facility managers present. In Bojanala, the workshop was attended by district managers only. Facility managers were engaged individually through on-site visits conducted by study staff prior to any training activities and introduction of the counsellors into the facilities. These visits orientated the facility staff to the intervention components as well as attempting to generate buy-in. An intervention coordinator supported each facility in the coordination and delivery of the different components on-site, especially the APC mental health training, forming the link between Department of Health particularly the Regional Training Centre (Departmental body responsible for in-service training) and the study staff and closely monitoring implementation. This additional support was deemed necessary given that Bojanala was not an NHI pilot site and, as such, did not have the same level of resources and support for APC trainings as Dr KK, where APC roll-out was implemented as part of the NHI piloting process.

#### Reach,adoption and implementation of cascaded APC training and clinical communication skills training

Training coverage of PHC nurses in the supplementary APC mental health module using the cascaded model of training was greater in Bojanala than in Dr KK, with adoption varying across facilities in both Dr KK and Bojanala. In Dr KK, the average exposure rate to one or more sessions across the facilities was 59%, (range: 36%-80%). The average rate for completed training across facilities was 39% (range: 0%-80%). In Bojanala, the average exposure rate to one or more sessions across facilities was 85% (range: 56%-100%). The average completion rate was 78% (range: 43%-100%).

While clinical communication skills training reached all the intervention facilities in both sites, exposure rate to one or more sessions across the facilities was 56% in Dr KK and 52% in Bojanala. However, the application of clinical communication skills when using the APC guide was strengthened in Bojanala in response to feedback from nurse clinicans in Dr KK for greater integration of the trainings. In this regard, the use of different clinical communication skills were signposted during the training of the APC clinical content. Furthermore, use of counsellor referral forms was also integrated into the APC training in Bojanala, so as to optimize confidence in making referrals.

#### Reach and implementation of the training for doctors

The training provided to primary health care doctors was provided by project employed psychiatrists, doctors and nurses. While doctors were mostly available on a sessional basis in both sites, they were more present in the PHC facilities in Dr KK, given that additional doctors were employed in the district through the NHI pilot. There was thus greater restrictions on the availability of doctors for in-service training in Bojanala. This contextual variation led to variations to exposure and content of the training across the two sites. In Dr KK, the training reached 65% of PHC doctors, who were exposed to more wide-ranging information on assessment, diagnosis and psychotropic treatment beyond CMDs, using both mhGAP and APC guildelines. In Bojanala, training reached just over 50% of the estimated number of PHC available. Further, they were only exposed to assessment and treatment of depression and anxiety using APC given greater time constraints.

#### Reach and implementation of counselling intervention training and supervision

All lay counsellors received the same initial offsite training as they were project employed. Weekly group supervision attendance was high across both district sites – with an average attendance rate by counsellors of 79% in Dr KK and 88% in Bojanala. However, group supervision in Bojanala was mostly online, whereas in Dr KK it was face-to-face.

Further variation across the sites occurred with respect to implementation of in-vivo supervision. Being better resourced and geographically smaller, all ten intervention facilities in Dr KK received in-vivo supervision, either from intern psychologists from the existing health system or a project-employed psychologist/registered psychological counsellor. However, in Bojanala only project-employed Bachelor of Psychology psychological counsellors were available to provide in-vivo supervision and fidelity assessments – as there were no psychologists/intern psychologists available to provide this service in the district. Further, given large distances between facilities, the use of audio-recordings for supervision were used more widely in Bojanala. Recordings were submitted to the supervisor during weekly group supervision. The supervisor listened to them aided by the fidelity checklist and provided feedback to the counsellor during their next meeting. In addition, due to the traumatic nature of the cases the counsellors were receiving in Bojanala, additional online psychological services were enlisted to provide debriefing for both the lay counsellors as well as the psychological counsellor supervisor. The services of a non-government organization (NGO) to provide forensic services and inter-personal violence (IPV) counselling in Bojanala to assist with the large number of disclosures of trauma, particularly rape and other violent crimes were also enlisted.

#### Reach, adoption and implementation of the counselling intervention

A total of 4298 patients were referred to the co-located counselling service across both districts, with 2907 of these in Bojanala over an 18 month period and 1391 in Dr KK over a 17 month period. Just under half of referred patients attended at least one session (*n* = 2200), with 6418 counselling sessions provided in total. Adoption of counselling sessions was higher in Dr KK where referred patients received an average of 3.61 counselling sessions relative to those in Bojanala who received an average of 1.55 sessions.

Counselling fidelity assessments were done in both Dr KK and Bojanala, whereby audio recordings of the counselling sessions were independently rated by two psychological counsellors with a Bachelor in Psychology qualification. In Dr KK, fidelity assessments for six or more session were done for 11 lay counsellors, with intervention fidelity ranging from 46 to 99% with an average of 71%. In Bojanala, seven lay counsellors received fidelity assessments for six or more sessions. Intervention fidelity ranged from 43 to 76% with an average of 61%.

## Discussion

Differences in implementation between the two trial study sites may have impacted implementation outcomes across the two sites, influencing the real-world effectiveness of the implementation package at the different sites. The importance of modifying system strengthening interventions to suit varying contexts in pragmatic trials that span more than one district is highlighted; as is key stakeholder involvement in co-designing district-specific implementation strategies to support implementation.

While a formative phase engaging in such processes was conducted in Dr KK, the same process was not applied in Bojanala, with the collaborative care model not adapted to suit the Bojanala context. As a consequence, system support for implementation of the PRIME/CoBALT package was not as forthcoming in Bojanala as it was in Dr KK. Engagement of key stakeholders in the co-design of systems strengthening interventions is vital in identifying potential challenges and solutions to promote uptake of innovations in real-world settings [41]. Further, layering interventions into the existing health system organizational structure and leveraging existing processes and available health resources assists to promote ‘goodness of fit’, enhancing the long-term potential of uptake and broader dissemination of the innovation [42].

Despite the model not being initially adapted to the Bojanala context, modifications in implementation of the package were made in response to these contextual differences; with some modifications also in response to learnings from implementation in the Dr KK site. An important contextual difference between the two sites was that Bojanala was much larger geographically than Dr KK, with vast distances between PHC facilities. Further, Dr KK was better resourced, especially with regard to PHC doctors and APC training support, both of which were introduced as part of the NHI piloting process. Bojanala, not being a NHI pilot site, was characterized by weaker human resource support (PHC doctor availability, APC training as well as specialist mental health services). Greater project support was thus provided in Bojanala. This was in respect of implementation and uptake of the Mental Health APC component of the intervention package developed for the Dr KK site, as well as mental health specialist support for the supervision and debriefing of the lay counsellors.

Greater project support for the APC component may have influenced the higher completion of the APC mental health training observed in Bojanala compared to Dr KK. This may in turn, have positively influenced the greater number of referrals made to the lay-counsellors in Bojanala, compared to Dr KK during the trial periods. Evidence suggests that in-service mental health training of PHC nurses is a key driver of referrals made [43].

Greater project support for the lay counselling intervention did not, however, translate into greater counselling uptake. While mental health specialist support for the supervision and debriefing of the lay-counsellors was entirely project supported in the Bojanala district, it was not greater than in Dr KK. In Dr KK, lay counsellors received a greater amount of specialist in-vivo individual supervision and support. In Bojanala, such in-vivo supervision and support for lay counsellors was not always possible because of long geographical distances and lay-counsellors were more likely to have had to record sessions with feedback provided on the recorded sessions. Further, Dr KK also had the advantage of having face-to-face individual emotional debriefing support provided by district intern psychologists, while in in Bojanala, this was provided remotely in groups via digital technology by project-employed psychologists. While other contextual factors may have influenced the lower uptake of counselling sessions by referred patients in Bojanala, greater in-vivo and face to face supervision and emotional support for lay counsellors observed in Dr KK may have influenced counsellor competency, with fidelity ratings lower in Bojanala. Fidelity has been previously found to impact on the uptake and effectiveness of lay-counselling services [37].

Since completion of the trials, the intervention has been refined and expanded for implementation and scale-up across different contexts using implementation science to understand the modifications required for the different contexts through the Mental health INTegration (MhINT) and Southern African Mental health INTegration (SMhINT) projects in KwaZulu-Natal (KZN) province of South Africa [44, 45]. Key elements that have been added to strengthen the intervention have been efforts to enhance the fidelity of APC mental health training, and the introduction of screening and psychoeducational demand generating components [46, 47] along the cascade of care for other conditions within the system.

## Conclusion

In the context of the large treatment gap in South Africa [5], the large number of patients referred to the co-located counselling service in the 10 facilities over the trial period of 17–18 months in each site is testament to the utility and acceptability of lay-counselling services as part of a collaborative care model within PHC settings in South Africa. However, the appropriateness of a one-size fits all collaborative care model for different contexts in South Africa was challenged. The importance of co-design of collaborative care models for in-country contextual differences, as well as in-country ‘implementation heavy’ research to adapt innovations for different contexts is highlighted. Innovations within health systems that accommodate varying contexts are more likely to find fertile ground in advancing efforts at scale-up.

## Supplementary Information

**Additional file 1.**

**Additional file 2.**

**Additional file 3.**

**Additional file 4.**

## Footnotes

1 2015 Health Workforce Normative Guides and Standards for Fixed PHC Facilities, Department of Health, South Africa.

2 Registered counsellors are mid-level psychological providers. They have a 4-year Bachelor Psychology (B.Psych) degree and registered with the Professional Board of Psychology in South Africa.

## Declarations

### Data availability

The anonymized data will be made available upon request in accordance with the PRIME publication and data management policies available at https://bit.ly/ 2tXQQsV. The data may be requested via the online expression of interest form at http://www.prime.uct.ac.za/contact-prime or through the Principal Investigator at Peterseni@ukzn.ac.za.

### Patient and public involvement

Patients were interviewed in the formative phase of the PRIME study to inform the development of cultually and contextually appropriate mental health care services at PHC. Additional interviews were conducted with patients in the PRIME cohort study to ascertain their experiences of the counselling intervention, and their suggestions to improve the services.

ORCID iD: Inge Petersen https://orcid.org/0000–0002-3573–4229.

Lara Fairall https://orcid.org/0000–0002-7460–4670.
