# Supplementary material for: A collaborative care package for depression comorbid with chronic physical conditions in South Africa
Source: BMC Health Serv Res. 2022 Dec 1;22:1465. doi: 10.1186/s12913-022-08874-7 (PMC9717432; doi:10.1186/s12913-022-08874-7)
Supplement: Supplementary file 1 — Additional file 1. [file 12913_2022_8874_MOESM1_ESM.pdf]

Supplement 2: TIDieR framework for Supplementary Adult Primary Care (APC) Mental Health Training of Facility Trainers

1. Brief name	APC Mental Health Training
2. Why	Given that the Basic APC training provided limited training in mental health, with only two out of the 27 cases focused on mental health, supplementary training was provided in the form of a focussed APC Mental Health Module to better equip nurses to use APC to screen for and assess, advise and refer appropriately any patient with a common mental health condition. The objective of the training was to increase health workers' confidence and ability to identify common mental health conditions and provide brief interventions and referrals presenting to primary care. The Mental Health module comprised four additional onsite sessions on mental healthcare, using nine mental health case scenarios. These scenarios focused on patients with various conditions designed to increase the users' confidence in diagnosing mild depression; diagnosing and treating moderate-severe depression; diagnosing and treating depression in a patient with other chronic conditions, including HIV; dealing with non-adherence to treatment due to depression; managing a suicidal patient; diagnosing and managing substance abuse; and managing a depressed patient who was not responsive to treatment. The training also highlighted the distinction and relationships between social stressors, especially poverty, and depression, as pilot work had showed that nurses tended to prioritise referral to social welfare ahead of providing clinical care for people presenting with depression (1) The training also addressed the stigmatised nature of mental illness, providing a non- judgemental space in which to speak candidly about working, and living with, people with mental illness.<br><br>During the formative phase of PRIME, the need for an orientation to the changes in the health care system, particularly the Integrated Clinical Services Management model as well capacitation in person-centred care was identified as was the need to strengthen capacity for providers to cope with their own underlying personal issues and the ability to communicate with patients on issues pertaining to their mental health (1). To complement the supplementary APC mental health module described above, a Clinical Communication Skills building training program was therefore developed to orientate nurses to the various systems changes happening, to understand their role within the new system, provide them with clinical communication skills necessary to for person-centred care that takes into consideration the emotional and physical well-being of a patients, as well as providing them with skills to cope with emotional labour.
3. What - materials	APC<br>Master Trainers materials:<br>1. APC Mental Health Training programme<br>2. APC Mental Health Facility Trainers' manual (described below) contained the training content<br>3. APC Guide<br>Facility Trainers' materials:<br>1. APC Guide<br>2. APC Mental Health Facility Trainers' Manual outlining the PRIME/COBALT intervention, the format for on-site training, and the session programme for each session. It contained the case scenarios of patients with various conditions designed to increase the users' confidence in diagnosing mild depression, diagnosing and treating moderate-severe depression, diagnosing and treating depression in a patient with other chronic conditions, including HIV, dealing with non-adherence to treatment due to depression, managing the suicidal patient, diagnosing and managing substance abuse, and managing the depressed patient that is not responsive to treatment.<br>Facility Staff materials:<br>1. APC Guide to be used in the on-site training and in consultation with patients

	Clinical Communication Skills<br>Onsite Training Manual, which was a step by step instruction manual for use by the facilitators when conducting onsite sessions at the clinic. Four sessions focused on:<br>• Session 1: Understanding and Dealing with Change: This session introduced participants to the complexities and dynamics of the consultation process and allowed them to engage with and discuss the changes that had taken place in the South African health care system, particularly the Integrated Clinical Systems Management model. Specific emphasis was made on how these system level changes demanded a more person-centred approach to integrated care for chronic illnesses and how this new approach benefitted both the health care provider and the patients.<br>Session 2: Gathering Holistic Information: This session introduced participants to taking a history from both the biomedical perspective and the patient perspective. The goal was to orientate the participants towards consulting patients in a more person-centred way that encouraged empowered and motivated patients.<br>• Session 3: Dealing with Emotions: This session dealt with addressing and containing emotions that came up during consultation with a patient. The session equipped the healthcare practitioners with the skills needed to help contain patient emotions during a challenging consultation as well as explored ways to deal with their own emotions and care for themselves to prevent burnout.<br>• Session 4: Motivation for Patient Self-Management: In this session the focus was on motivational interviewing skills that helped the healthcare practitioner to motivate for change in their patients to reach the goal of a self – managing activated patient. This session also aimed to capacitate participants with the skills needed to develop a self – management plan collaboratively with their patients.
4. What - procedures	APC<br>The Facility Trainers attended a workshop that built on the Basic APC training and format of on-site sessions but focused on strengthening the mental health component of APC.<br>The Facility Trainers then cascaded the training in 4 on-site training sessions to their colleagues at their facilities.<br>Clinical Communication Skills<br>Project staff arranged meetings with each facility manager to discuss the training as well as arrange for dates when project staff can return to conduct the trainings. Clinics had the autonomy to decide when would be convenient for the trainings to happen so as not to interrupt service delivery
5a. Who provided	APC<br>KTU Master Trainers trained APC Facility trainers from the selected facilities.<br>The Facility Trainers in turn trained the staff in their facilities.<br>Clinical Communication Skills<br>The intervention was provided by project employed facilitators who had a Master's degree in Psychology and who had undergone training and mentoring related to facilitating CCS workshops

	The facility staff used APC and Clinical Communication Skills to consult patients in a person-centred standardised manner, specifically regarding screening, assessing, diagnosing, managing and referring patients with common mental health conditions.
5b. Who benefits	The nurses benefitted from having access to the latest clinical policies and recommendations as laid out in APC. This was intended to build confidence (2) and create a standardized approach to care and treatment (3), specifically with regards to the integration of common mental health conditions (4).<br><br>The patients benefit from staff using APC and Clinical Communication skills as, it was anticipated that more chronic care patients would be screened, assessed, diagnosed and managed for depression and substance abuse in addition to their presenting medical condition.

6. How	The APC Mental Health Module Facility Trainers' workshop provided:<br>• A structured four-step approach to on-site training<br>• The opportunity to engage with the clinical content focusing on strengthening the integration of mental health screening, diagnosis, and management of chronic care patients presenting with symptoms of comorbid common mental disorders, including appropriate referrals.<br>• The opportunity to practise facilitation of the case scenarios prior to on-site delivery of the training themselves.<br><br>The APC Mental Health Module on-site training sessions:<br>• Built on the use of case scenarios featuring the common mental health conditions, as well as focused on the collaborative care model, clinical communication skills and the referral pathways available to support patients.<br><br>The Clinical Communication Skills workshops were delivered in a group format, adopted adult learning principles, were interactive and participative, and included role plays and practising of CCS skills by the participants<br><br>Facility Staff were better equipped to use Clinical Communication Skills and reference APC during a consultation with a patient to ensure that:<br>• They were giving up-to-date, person-centred policy aligned care and treatment.<br>• They were using clinical communication skills during their consultations<br>• They were referring patients with a common mental disorder appropriately within the PRIME/CoBALT collaborative care model depending on symptom severity.
7. Where	The APC Mental Health Facility Trainers workshop:<br>The training in both Dr KK and Bojanala districts took place at a central off-site venue that was accessible to the participants.<br><br>The APC Mental Health on-site and Clinical Communication Skills training sessions:<br>These sessions were run at the facilities to prevent staff having to take time away for off-site training.<br><br>Use of APC and Clinical Communication Skills:<br>In consultations with patients.
8. When and how much	The APC Mental Health Facility Trainers' workshop:<br>In Dr KK this training was a 2 ½ day workshop.<br>In Bojanala the training was run twice as a 2-day workshop to stagger implementation (first training for the first five clinics, second training for the second five clinics).<br><br>The APC Mental Health on-site training sessions:<br>The four mental health sessions were run on-site. Three sessions at weekly intervals over one month, and the fourth session a month to assess implementation in the clinic. All professional nurses in the facilities were eligible for training.<br><br>Clinical Communication Skills:<br>Four on-site sessions facilitated by two CCS trainers<br><br>Use of Clinical Communication Skills and APC:<br>As an approach and reference tool in consultations by a health worker with a patient.

9. Tailoring	The training of the second group of Facility trainers in Bojanala in the APC Mental Health module was shortened to two days as all Facility Trainers had recently attended a Basic APC Facility Trainers' workshop, and so were up to date.

10. Modifications	In Dr KK the Clinical Communication Skills was provided on-site directly to the nurse clinicians by Clinical Communication Skills trainers. This training was cross-referenced during the APC Mental Health training, to ensure that participants were aware that it was part of the same intervention.<br><br>In Bojanala the Clinical Communication Skills material was fully integrated into the APC Mental Health training following feedback from the nurse clinicians in Dr KK on the need to integrate these trainings and be trained in the same way using the cascade model of training. The APC cases featured information and discussion points to build Clinical Communication Skills alongside the clinical content. In the first group, the Clinical Communication Skills component was facilitated by a Clinical Communication Skills trainer from the University of KwaZulu-Natal.<br>The KTU Master Trainers took on this Clinical Communication Skills training when the second group was trained.<br><br>In Bojanala, the counsellor referral forms to be used within the collaborative care model were featured in the training to ensure confidence in how to use them for appropriate referral.
11. How well - planned	APC Mental Health Facility Trainers workshop:<br>Training was assessed using pre- and post-training evaluation questionnaires and perceived confidence in using APC.<br><br>APC Mental Health on-site training sessions:<br>Training coverage was assessed by tracking the proportion of the four sessions successfully delivered to staff at participating facilities.<br><br>The Clinical Communication Skills training programme planned to have all nurses in the intervention facilities trained in all four sessions
12. How well - actual	APC Mental Health Facility Trainers' workshop:<br>In Dr KK, eight out of the 10 intervention clinic Facility Trainers attended.<br>In Bojanala, all ten Facility Trainers attended the training sessions.<br>The training was well received, with nurses commenting in the training evaluations that their awareness and appreciation for mental healthcare had increased as reflected in some quotations from this evaluation below:<br>• *"The training was as eye opener as mental health care users were overlooked before, e.g. clinicians just concentrated on their monthly routine follow-up, forgetting that they may develop other conditions that need medical attention."*<br>• *"The gap which I realised is that I did not know that poor adherence can be due to depression or mental health problems. In the process of training no gap was identified, but my personal feeling is that this training should have started years ago, lives of other patients would be saved long ago, but now we have lost lives. Please continue this training to save lives."*<br><br>APC Mental Health on-site training sessions:<br><u>Dr KK</u><br>All ten intervention clinics initiated the APC Mental Health training. A KTU master trainer provided the on-site training sessions in the two intervention facilities which did not have Facility Trainers attend the APC Mental Health Facility Trainers' workshop. Of the total number of nurses (148), 66% were exposed to at least one session. The average exposure rate was 59% (range: 36-80). Fifty-two (35%) were fully trained, with the average rate for completed training being 39% (range: 0-80). See Figure 2.1 for a breakdown per facility. The majority of training sessions were completed prior to the 6-month primary outcome follow-up period.<br><br>Clinical Communication Skills<br>Based on training reports, nurses were exposed to one or more sessions in all 10 intervention clinics in both Dr Kenneth Kaunda and Bojanala districts. In total 83 nurses were exposed to CCS training in Dr KK and 73 nurses in Bojanala. Feedback on the training sessions suggest that the training was perceived as helpful for promoting person-centred care.<br>• *"Communication skills was very interesting because it really built my behaviour and attitude not only towards to the patient but also to anybody."*

	• *"The process has taught me a lot about how I should approach my patient. Attitude we give to patients can make them not to be adherent."*<br>• *"The better way of handling or managing patients using PRY, ICE, NURS and the 5A's. The fact that we are going to have behaviour change counsellors, I think they will assist us and the patients a lot*

Figure 2.1: Dr KK Facility-based APC Mental Health training coverage

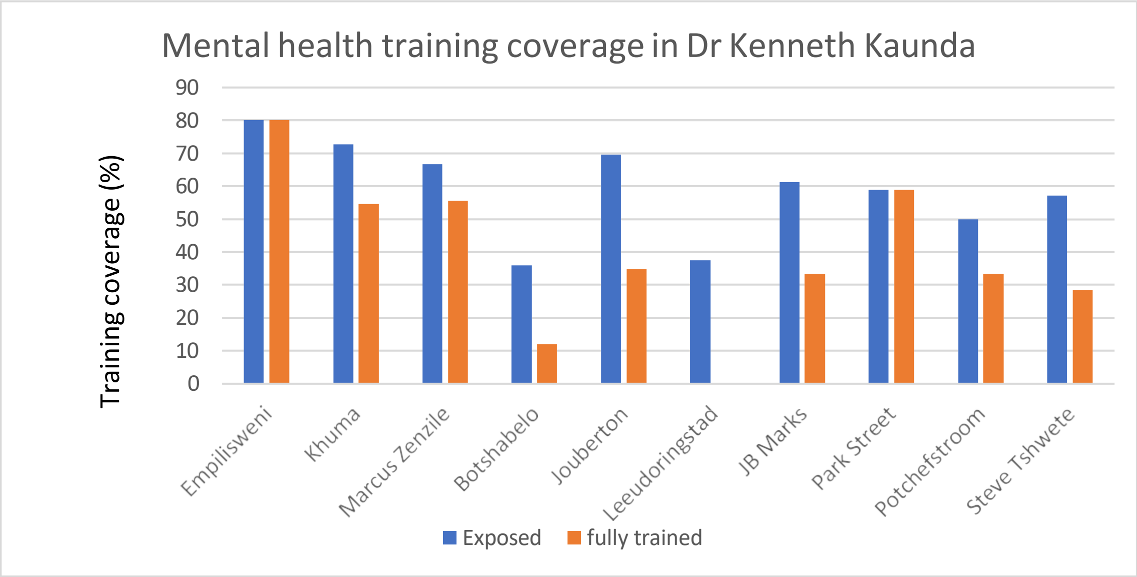

Bojanala:
All ten intervention facilities initiated APC Mental Health training. The average exposure rate was 85% (range: 56-100%). The average completion rate was 78% (range: 43-100%) which was higher than Dr KK. (See Figure 2.2 for a breakdown per facility). However, one sixth of the training sessions were completed following the 6-month primary outcome follow-up period.

Figure 2.2: Bojanala facility-based APC Mental Health training coverage

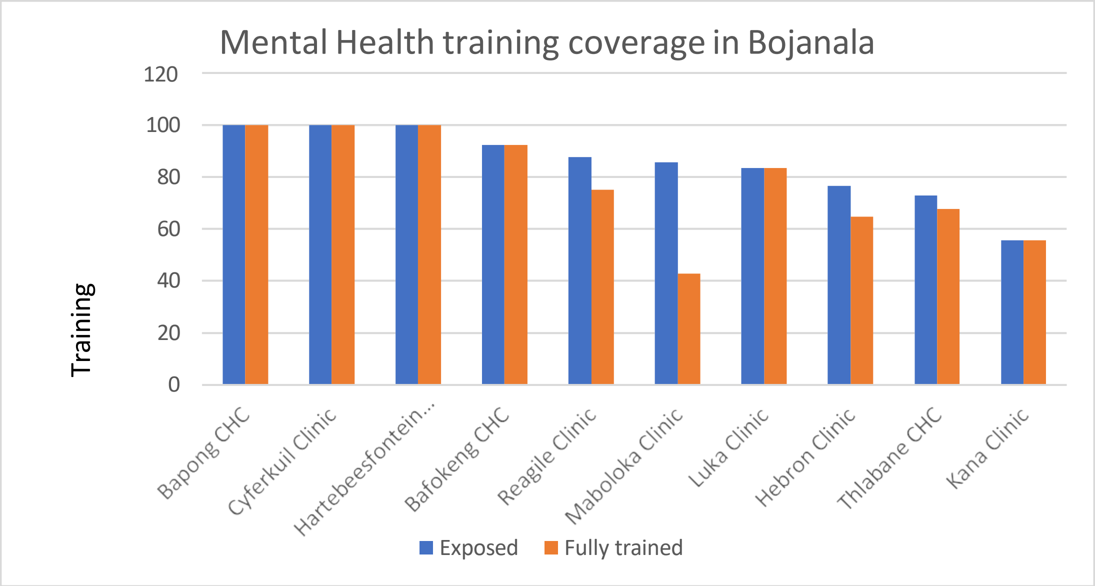

Over the duration of the counselling intervention, nurses trained in the APC Mental Health Training referred 4298 clinic patients to counsellors based at the clinic. There were more referrals in Bojanala (n=2907), compared to DKK (n=1391). Of the total referred, 2200 attended at least one session. In total, 6418 counselling sessions were provided. However, only 137 (3%) of the referred patients were enrolled in the PRIME and CoBALT trials.

13. Voice – who was involved	The Knowledge Translation Unit training and implementation team, the clinical team, the research team.<br>The Department on Health Regional Training Centre<br>UKZN research team

References

1. Petersen I, Fairall L, Bhana A, Kathree T, Selohilwe O, Brooke-Sumner C, et al. Integrating mental health into chronic care in South Africa: the development of a district mental healthcare plan. Br J Psychiatry. 2016;208 Suppl 56:s29-39.
2. Georgeu D, Colvin CJ, Lewin S, Fairall L, Bachmann MO, Uebel K, et al. Implementing nurse-initiated and managed antiretroviral treatment (NIMART) in South Africa: a qualitative process evaluation of the STRETCH trial. Implementation science : IS. 2012;7:66.
3. Stein J, Lewin S, Fairall L, Mayers P, English R, Bheekie A, et al. Building capacity for antiretroviral delivery in South Africa: a qualitative evaluation of the PALSA PLUS nurse training programme. BMC health services research. 2008;8:240.
4. Simelane ML, Georgeu-Pepper D, Ras CJ, Anderson L, Pascoe M, Faris G, et al. The Practical Approach to Care Kit (PACK) training programme: scaling up and sustaining support for health workers to improve primary care. BMJ Glob Health. 2018;3(Suppl 5):e001124.
