# Supplementary material for: A collaborative care package for depression comorbid with chronic physical conditions in South Africa
Source: BMC Health Serv Res. 2022 Dec 1;22:1465. doi: 10.1186/s12913-022-08874-7 (PMC9717432; doi:10.1186/s12913-022-08874-7)
Supplement: Supplementary file 2 — Additional file 2. [file 12913_2022_8874_MOESM2_ESM.pdf]

Supplement 4: TIDieR framework Mental Health Doctors workshop

<table>
<tr><td>1. Brief name</td><td>Doctors workshop (Mental health focus)</td></tr>
<tr><td>2. Why</td><td>Initial workshops in Dr Kenneth Kuanda (Dr KK) district focused on mental health care for adults attending primary care facilities. The objectives of the training were:
o to provide an approach to screening and assessment of mental disorders in adults attending primary care facilities
o to increase capacity of Primary Care Doctors to manage mental disorders, including through initiation and monitoring of psychotropic medication

The aim of the training in the Bojanala district was to orientate the doctors to the PRIME/CoBALT trial and their role in providing mental health care. The workshop did not cover in-depth clinical mental health content as the aim was to show that you don’t need to be a psychiatrist to treat common mental disorders. Objectives included the following:
o Orientation to the APC Guide, with a specific focus on the mental health section.
o Orientation to the collaborative model of care
o Orientation to the trial
o The importance of managing mental health conditions at Primary Health Care (PHC) level, including initiating psychotropic medication when indicated
o Identify barriers to prescribing antidepressants</td></tr>
<tr><td>3. What - materials</td><td>In the workshops held in Dr KK, the following materials were used:
• mhGAP intervention guide
• APC guide
• Powerpoint presentations and handouts
• Case material covering:
  o Crisis/adjustment disorder
  o Generalized anxiety disorder
  o Panic disorder
  o Post-traumatic stress disorder
  o Depressive disorder

In Bojanala the following material was used:
• APC guide
• APC mental health aligned introductory activity
• APC case scenarios covering the following:
  o Using symptoms to prompt the screening and diagnosis of depression
  o Using the stressed or miserable patient page in APC to manage mild depression and anxiety
  o Initiation of antidepressant medication for moderate severe depression and follow-up monitoring of response to medication.
  o Using clinical communication skills
• Powerpoint Presentation describing the overview of context and reasons for taking mental health seriously: No health without mental health is presented as 10 reasons why we cannot ignore Mental Health:
  1. Common, very common.
  2. Associations with physical illness
  3. Somatisation and mental health as the underlying “invisible illness”
  4. Potential to compromise adherence
  5. Size of the treatment gap</td></tr>
</table>

	6. Impact of mental disorders on poverty – and the potential for addressing mental disorders to liberate people from poverty<br>7. Contribution of mental disorders on health system burden<br>8. Effective treatment available<br>9. Effective treatment cheap<br>10. Legislation means only doctorss can prescribe in our system
4. What - procedures	In Dr KK there was an initial two-day interactive workshop which was followed up by a half-day workshop.<br>In Bojanala two four-hour facilitated workshops with interactive discussion and presentation of information.
5a. Who provided	Workshops provided in Dr KK were delivered by a community psychiatrist, with a special interest in integrating mental health into primary care and HIV and mental health.<br>At each workshop in Bojanala there was at least one doctor and one nurse facilitator from the Knowledge Translation Unit (KTU). Both doctors were experienced in primary care, one being a family physician, and both were involved in the development of APC guide. Three of the KTU's nurses co-facilitated the workshops. One of the nurses leads the Implementation and Training team and has a background in primary care and management. The second nurse has extensive training experience and a particular interest in mental health and its integration into care. The third nurse was based in Bojanala for the duration of the trial and had an in-depth understanding of the clinic context and support needed by the nurses. She provided follow up support at a facility level.
5b. Who received	Doctors working in the Primary Heath Care facilities that provided services at intervention clinics.<br>Many of the doctors worked at several clinics on a sessional basis and therefore provided care at both intervention and control clinics.
5c. Who benefits	The doctors themselves benefitted as they were provided with information and tools to treat patients presenting at primary care facilities with common mental health illness. Many of the doctors had initially expressed a discomfort with prescribing antidepressants and signalled that the training had helped to increased their confidence in this regard. In the workshops done in Dr KK a pre- and post-training assessment of knowledge was done which showed an overall increase in knowledge (70% average pre-training and 80% average post-training).<br>Nurses were expected to benefit from the training through potentially receiving additional support from the doctors in managing more complex cases.<br>Patients should potentially benefit through better care.
6. How	An introductory activity was used to highlight the mental health content of the APC guide. This was followed by use of case scenarios of patients with chronic conditions including co-morbid mental health conditions. The aim was to build confidence in users to access the correct information in the APC guide for integrated assessment, advice and treatment of presenting symptoms or conditions. These sessions were interactive and facilitated by the KTU facilitators.<br>A session was done to identify barriers to prescribing antidepressants and a presentation was given on reasons for taking mental health seriously.
7. Where	Workshops took place at Department of Health boardrooms in Dr KK and Bojanala districts.

8. When and how much	In Dr KK there was an initial two-day workshop with 15 doctors in attendance out of a total of 23 (65% coverage). In Bojanala there was an initial half-day workshop attended by nine doctors; with a follow-up half-day workshop attended by 12 doctors out of an estimated total of 20 doctors* (just over 50% coverage)
9. Tailoring	The following feedback for future tailoring of the intervention was received from the doctors' workshops in Bojanala:<br><br><u>Barriers to prescribing antidepressants</u>:<br>o Poor availability of antidepressants especially fluoxetine and citalopram at Primary Health Care facilities.<br>o Minimal diagnosis of depression – with most patients being prescribed 25mg amitriptyline for insomnia rather than fully assessed for a diagnosis of depression.<br>o No standardized screening instrument for depression at Primary Health Care facilities.<br>o Initiation of antidepressant medication dependent on doctors prescribing. Some doctors' time was sessional, with most of their time at the clinics consumed by complex HIV and TB cases, leaving limited time to attend to mental health cases.<br>o Doctors also perceived antidepressants to be high cost. In reality at the time of training fluoxetine cost ZAR3.54 per month, citalopram ZARR6.94 a month and amitriptyline ZAR7.89 per month (all well below 1USD/ month).<br>o There were mixed reactions from the doctors to support telephonic prescriptions of antidepressants.<br><br><u>Items that they requested additional support with:</u><br>o Management of acute psychosis<br>o When and how to stop antidepressants<br>o How long to continue antidepressants<br>o How to manage substance use<br>o Effective psychological treatments<br>o Refresher training in the Mental Health Care Act<br>o Psychosocial support for chronic psychotic patients<br>o Paediatric psychiatry<br>o Suicidal risk assessments
10. Modifications	While the training in Dr KK covered both severe and common mental illness, the focus in Bojanala was on equipping doctors to use resources and tools to manage common mental health illnesses.
11. How well - planned	No formal evaluations of the workshops were planned to be carried out.
12. How well - actual	The second workshop in Dr KK workshop was very poorly attended because there was confusion regarding times, and eight doctors arrived at 8am in the morning when it was only scheduled for 12 noon. Only three doctors could come back for the noon session but subsequently one of them has presented the slideshow and discussed the content at their regular doctors' meeting.
13. Voice – who was involved	o Knowledge Translation Unit<br>o Principal Investigator of the Africa Focus on Intervention Research for Mental health<br>o Professor in the Department of Psychiatry, Faculty of Health Sciences, University of the Witwatersrand

	○ Centre for Rural Health (CRH) in the College of Health Sciences and Professor of Psychology at the University of KwaZulu-Natal

*Estimated from the 2015 Health Workforce Normative Guides and Standards for Fixed PHC Facilities
