# Supplementary material for: A collaborative care package for depression comorbid with chronic physical conditions in South Africa
Source: BMC Health Serv Res. 2022 Dec 1;22:1465. doi: 10.1186/s12913-022-08874-7 (PMC9717432; doi:10.1186/s12913-022-08874-7)
Supplement: Supplementary file 3 — Additional file 3. [file 12913_2022_8874_MOESM3_ESM.pdf]

Supplement 4: TIDieR Template for the lay counsellor intervention including training and supervision

**1. Brief name**	**Task-shared counselling for depression**
**2. Why**	The goal of the lay counsellor training and supervision was to equip counsellors to enable them to deliver a task-shared eight session manualised depression counselling intervention with an additional adherence counselling session. Formative work identified poverty; interpersonal conflict; social isolation/ avoidance; grief and bereavement; internalised and externalised stigma as casual factors for depression (Selohilwe et al., et al., 2019). The intervention was made up of eight sessions and an additional adherence session called "Getting to know your chronic condition(s) and medication". Six sessions were centred around addressing each of the identified triggers and issues that maintain depressive cycles using micro-counselling skills (active listening, reflection, clarification), and drawing on adapted Cognitive Behavioural Therapy (CBT) techniques, including problem solving, healthy thinking and behavioural activation, as well as grief and bereavement counselling. A psychoeducation session was used to introduce service users to depression and the last session was a closure session.
**3. What – material**	Training was designed to mirror the counsellors' activities when delivering the intervention using the intervention materials they would use when providing the service. These included:<br><br>1. Waiting Room Educational Talk script<br>The waiting room educational talk was designed to help adult patients to identity and report their symptoms to the consulting health worker.<br><br>2. Resource manual<br>This manual was used to help lay counsellors prepare for their sessions. The manual provided session examples and potential challenges as well as how to deal with the challenges in the session challenges.<br><br>3. Counselling Manual for Depression: The Lay counsellor's guide to depression<br>This manual was organised into the following sections:<br>i. Introduction to depression<br>This was a psychoeducation session offered to all patients referred to the lay counsellor during their first meeting which took place on the day of the referral.<br>ii. Leading groups<br>This was an 8-session step by step intervention manual which lay counsellors used when facilitating group counselling

sessions. Six of the sessions were centred around each of the identified triggers and issues that maintain depressive cycles (poverty; interpersonal conflict; social isolation/ avoidance; grief and bereavement; internalised and externalised stigma). The last session was a closure session.

iii, Individual counselling

This manual was structured in a similar manner to the Leading Groups manual above with a focus on individuals.

iv. Getting to know your chronic condition(s) and medication

There was an additional session to the abovementioned eight sessions that focused on facilitating adherence counselling and providing accurate information on chronic conditions and patients' prescribed treatment where needed through the use chronic conditions educational pamphlets.

4. Chronic conditions educational pamphlets

These are short leaflets (half an A4 page) containing some information on the condition, purpose of treatment, what lifestyle changes patients could adopt to assist with maintaining their health, and when and how quickly to seek care. Leaflets were available in the dominant local language in the district (Setswana) on one side and English on the reverse. These have since been incorporated into a large volume on chronic conditions as part of a resource to assist people during the de-escalation of primary care services during the COVID-19 pandemic and can be found at: https://www.coronawise.org.za/

5. Intervention forms

• Intake assessment forms

These were used to capture patient's identifying information on the first day the patient met with the lay counsellor.

• Group booking forms

These were used to allocate patients who wanted group counselling to a group. The group was booked over a period of two weeks and patients seen over this time were allocated to the same group with a group capacity of eight to 10 people. The groups were gender specific.

• Group and individual attendance registers

These were used to keep record of patients' attendance. Patients that missed a session were followed up and offered a catch-up session.

• Confidentiality forms

Patients and the lay counsellor signed this form, thereby committing to maintaining confidentiality.

• Suicide referral forms

Patients who talked about wanting to die and/or had a recent attempt of suicide were referred to the consulting

	professional nurse/ doctor for further management with this form.<br>• Back-referral forms<br>These forms were used to refer patients who had completed their sessions or had expressed they did not want to continue with the intervention to the consulting professional nurse for re-assessment for depressive symptoms.<br>• Progress notes templates<br>These were used to keep record of patients' progress.<br>• Self-rating scale<br>These forms were part of the in vivo supervision process and the lay counsellor used them to assess his/her performance. Their answers were matched with their supervisor's and discussed.<br>• Weekly statistics forms<br>The lay counsellor used these forms to keep a record of all the patients he/she saw for the week. The form helped to inform the mental health statistics for the lay counsellor's PHC facility.<br>• Counselling referral forms<br>These forms were used by the Professional Nurse to refer patients to the lay counsellor.<br>• Patient feedback guide<br>This was used to help the patient to provide feedback about the intervention at the staff meeting.
**4. What – procedures**	Training was divided into three phases:<br>**Phase 1: Structured group training- 5 days**<br>This was informed by adult learning and teaching theories using experiential, interactive and reflective learning<br>**Phase 2: Peer to peer learning**<br>Counsellor trainees were placed with more experienced counsellors in their facilities allowing them to observe and practise their newly learnt skills.<br>**Phase 3: Apprenticeship training as well as continuous supervision and mentoring**<br>In the third phase the counsellors are placed in their designated facilities and received on-site in-vivo clinical supervision and participated in weekly group supervision meetings at a central location. Ad-hoc supervision was also provided when needed<br>**On-site in-vivo clinical supervision** involved a supervisor assisting the lay counsellor while he/she facilitated

	group/individual sessions. The supervisor's role was to model the correct way of running the session and deal with issues that may arise in the capacity of a co-facilitator so that the session itself was not disrupted. In addition, the supervisor observed how the counsellor facilitated the group and reinforced skills attained during training by providing feedback to the counsellor after the depression counselling session through the use of a fidelity checklist adapted from the ENhancing Assessment of Common Therapeutic factors (ENACT) rating scale*. Through this process, the lay counsellor thus not only observed and learned how to deal with similar issues in the future, but also received feedback that could help with facilitation of future sessions.<br><br>**Off-site group supervision** involved lay counsellors came together once a week creating a learning community where they reported on and received help with their administrative duties (for example providing statistics of patients referred); presented and discussed cases; shared their successes and challenges in the facilities allowing for group shared problem management, and received help for their emotional needs related to the intervention. The latter was added during the first month of the intervention in Dr KK and was formalised in Bojanala.<br><br>**Ad hoc supervision** involved counsellors contacting the supervisor whenever they needed to outside of the specified supervision time.
**5a. Who provided?**	Training was delivered by the overall project counselling intervention coordinator who was a clinical psychologist and co-facilitated by registered psychological counsellors, with a Bachelors in Psychology (B. Psych) degree who were fluent in Setswana and English. Supervision was provided by a sub-district psychologist, intern psychologists from the district hospital (Dr Kenneth Kaunda) and district and project-employed registered psychological counsellors who were oriented to their role as supervisors by the overall project counselling intervention co-ordinator.

**5b. Who received?**<br><br><br><br><br><br><br><br><br><br><br><br><br><br>**5c. Who benefited?**	Project employed lay counsellors who were selected with the following criteria:<br>• Grade 12 education (school leaving certificate)<br>• Knowledge of counselling and its diverse applications<br>• Maturity: emotional and intellectual – readiness to undertake 'emotional work'<br>• Relevant experience<br>• Willingness to provide depression counselling<br>• Insight into self, including own motivation<br>• Confidence<br><br>In Dr Kenneth Kaunda (Dr KK) 10 lay counsellors were initially trained all together and an additional six trained to replace those who got other jobs. Training was done in two consecutive phases in Bojanala with five lay counsellors being trained in each phase. An additional lay counsellor was trained to replace one who had left. A high lay counsellor turnover rate led to a total of 27 counsellors were trained over the trial period; 16 in Dr KK and 11 in Bojanala.<br><br>Lay health workers were capacitated with counselling skills for people identified with comorbid depression attending a chronic care clinic.<br>Patients benefitted from the counselling intervention as it was anticipated that more patients would have access to counselling
**6. How**	**Training and Supervision**<br>**Phase 1: Structured group training- 5 days**<br><br>The lay counsellors were brought together to learn as a group. They were orientated to the intervention package and their roles as part of the health care team in a primary health care facility. Training was designed to promote understanding of the different counselling techniques through role modelling of the different techniques by the trainers as well as providing opportunities to practice the different techniques in a safe space.<br><br>**Phase 2: Peer to peer learning**<br><br>Each trainee lay counsellor was placed with an experienced lay counsellor in a facility for five days where the trainee lay counsellor observed facilitation of counselling sessions and other intervention activities as well getting an opportunity to implement his/ her newly learnt skills. A peer-to-peer learning form was used to guide and record this process. This step

	was developed to train newly recruited lay counsellors replacing those who had resigned in Dr Kenneth Kaunda (Dr KK) district. It was formalised in Bojanala district.<br><br>**Phase 3: Apprenticeship training and continued supervision and support**<br><br>In the third phase the lay counsellors were placed in their designated facilities and received both on-site clinical supervision, involving observation of their counselling skills and feedback; as well as weekly group supervision.
**7. Where**	Training and supervision<br>Phase 1: Structured group training- off site at a training venue<br>Phase 2: Peer to peer learning- Primary health facilities<br>Phase 3: Apprenticeship training as well as continuous supervision and mentoring- Primary health facilities and offsite<br><br>Use of the manualized counselling intervention during counselling sessions
**8. When and how much**	Training and supervision<br>Phase 1: Structured group training- five days offsite<br>Phase 2: Peer to peer learning- five days onsite<br>Phase 3: Apprenticeship training as well as continuous supervision and mentoring onsite and offsite<br><br>Use of the manualized counselling intervention during counselling sessions
**9. Tailoring**	Training was tailored for individual lay counsellors who were recruited to replace lay counsellors who had resigned in Dr KK. These lay counsellors, who were often trained individually, received face to face training and to make up for the lack of group learning and to help foster their confidence, and were placed with more experienced lay counsellors to shadow them for five days. They were then placed in the designated facilities where they received continued supervision. The peer-to-peer learning process was formalised as Phase2 of training in Bojanala.
**10. How well – planned**	All lay counsellors were to receive training and supervision as planned.<br>Referred counsellors were expected to receive counselling as planned.

**12. How well – actual**	<u>Training and supervision</u><br>Dr KK<br>The first batch of 10 lay counsellors in Dr KK received structured off-site group training as well as in-vivo clinical supervision using the adapted ENACT rating scale, either from the intern psychologists or the project-employed psychologist/registered B.Psych counsellor. However, they were not exposed to the peer to peer learning. A total of eight counsellors from this initial batch resigned to take up more attractive positions with non-governmental organizations (NGOs) recruiting lay counsellors in the area, and one was redeployed during the intervention phase. New counsellors were consequently recruited and trained at various times. Two of these received individual training at two different times and four were trained as a group during the trial period in Dr KK. The off-site group/individual training and the apprenticeship training was bolstered for these new trainees through the introduction of peer-to-peer-learning whereby the new counsellors paired with the more experienced and competent counsellors in their clinics following the off-site training for a period of one week.<br><br>When supervisors were not able to provide in vivo supervision for each of the eight sessions, lay counsellors were asked to record these sessions for fidelity assessment by the supervisor. Fidelity assessment were done for 14 counsellors, with 11 counsellors receiving fidelity assessments for six or more sessions.<br><br>Fidelity assessment were done for 14 lay counsellors, with 11 of these receiving fidelity assessments for six or more sessions. Two B.Psych counsellors independently scored the audio recording and an average of the two scores was used as the final score.<br><br>Bojanala

	Lay counsellor training and supervision in Bojanala involved the structured off-site training, the peer-to-peer- learning stage, as well as the in-vivo clinical supervision using the adapted ENACT rating scale, thus incorporating all three training stages. An additional counsellor had to be trained individually off-site to replace one who resigned. The counsellor was trained in the same three phases. It became difficult to recruit and train more counsellors in both sites towards the end of the trial period with four facilities in Bojanala and three in Dr KK having to share a counsellor.<br><br>Supervision in Bojanala was provided by project-employed registered B. Psych psychological counsellors as no psychologist/intern psychologists available to provide this service in the district. They provided in-vivo supervision and fidelity assessments as well as group supervision. Due to the traumatic nature of the cases the counsellors were receiving, the need for a psychologist to provide debriefing was identified and an external psychologist was brought in to provide this service. An additional psychologist was also recruited to provide support and mentoring for the registered psychological counsellor.<br><br>Given large distances between facilities, the use of audio-recordings for supervision and fidelity assessment was more widely used in Bojanala. Recordings were submitted to the supervisor during weekly group supervision. The supervisor listened to them aided by the fidelity checklist and provided feedback to the counsellor during their next meeting.<br><br><u>Intervention quality and delivery</u><br><br>Patients who took up counselling in Dr KK received an average of 3.61 counselling sessions and those in Bojanala received an average of 1.55 sessions.<br><br>In Dr KK, fidelity assessment were done for 14 lay counsellors, with 11 of these receiving fidelity assessments for six or more sessions. Two B.Psych counsellors independently scored the audio recording and an average of the two scores was used as the final score. Intervention fidelity ranged from 46% to 99% with an average of 71%. In Bojanala, a total of 10 lay counsellors received fidelity assessments, with seven receiving fidelity assessments for six or more sessions. Intervention fidelity ranged from 43% to 76% with an average of 61%.<br><br>Qualitative process interviews were held with a stratified sample of patients: those who did not take up the counselling; those who attended one to four counselling sessions; and those who attended five to eight counselling sessions. These interviews revealed that patients did not take up the intervention for various reasons including: having to travel long distances to the facility (this was particularly the case in Bojanala); the lay counsellor not being available on the day of referral; not knowing what to expect from the counselling service; not receiving follow up dates; and wanting to leave the

	clinic after waiting for long periods before getting a consultation with the professional nurse. Patients reported dropping out before completing all the depression sessions because they felt better; had other social responsibilities or had found jobs and we not able to continue attending sessions.<br><br>Although space was a big challenge, the lay counsellors ensured patients' privacy during the counselling sessions. Lay counsellors shared counselling room space with other service providers where a dedicated space for counselling was not available. In one facility, counselling sometimes took place outside, behind the facility building away from the other patients' eyes. The facility yard was walled and this provided some level of privacy for the patients. Two facilities used the car garage, while a third initially used the sluice room. Although space continued to be a challenge throughout the trials, available counselling space improved with continued engagement.<br><br>Lay counsellors dealt with difficult cases that, sometimes, involved sexual violence and other crimes. While such cases were present in both districts, Bojanala lay counsellors experienced a higher prevalence of forensic cases. Lay counsellors were also exposed to traumatic information as patients relayed their stories during sessions. Again, Bojanala lay counsellors were exposed to a higher number of cases with trauma content. There was one clinic in Bojanala that was particularly affected, with the area served by the clinic without a police station or social services. The lack of these other services resulted in the lay counsellor receiving a lot of inappropriate referrals involving sexual violence and other criminal activities, which became a threat to her own emotional wellbeing. The lay counsellor was recalled from the facility for a period of two months, while services for the area from a non-governmental agency that specialized in such cases was sourced. The lay counsellor was provided with debriefing counselling by an external psychologist during this time which continued when she returned to her post. This debriefing counselling service was also subsequently made available to all the lay counsellors in Bojanala.
**13.Modification**	Training and supervision<br>Training was modified from two to three phases. Peer to peer mentoring, described above, was initiated in Dr KK to help with training of newly recruited counsellors replacing those who had resigned and was formalised in Bojanala. The final training phases are presented below:<br><br>A registered counsellor with a Bachelor Psychology degree replaced intern psychologists/psychologists as supervisors in Bojanala<br><br>Audio recording for fidelity assessments started in Dr KK and was used more widely in Bojanala

	Intervention delivery<br><br>When the counselling intervention was originally initiated in Dr KK, the lay counsellors were expected to follow the sequence of the sessions for the identified triggers as outlined in Figure 2. This was modified five months into the trial to allow for flexibility to respond to a patient's most pressing need first. The patient and lay counsellor worked together following intake and the "Understanding depression" session to identify the patient's most pressing need (poverty; interpersonal conflict; social isolation; grief and bereavement; internalized stigma or external stigma) which was then dealt with during the second session scheduled for the following week. The third session was then dedicated to "Getting to know your Chronic Condition and Medication(s)" where the patient would bring all their chronic medication to discuss with the lay counsellor. At this point, the patient was then also asked to choose between individual or group counselling. The patient then continued with the rest of the sessions as they were sequenced in Figure 1.<br><br>In order to help with increasing referrals received, lay counsellors were asked to make sure referral forms were available in designated areas in all the consulting rooms one month into the intervention. This followed an initiative by one of the lay counsellors which led to a high number of referrals in her facility.<br><br>Given that some patients dropped out of the intervention before completing all their sessions, patients who did not want to continue with the sessions were referred back to the consulting health worker for reassessment of depressive symptoms at this point rather than having to complete all sessions.
**13. Monitoring and coordination**	Training and supervision records were kept by the lay counselling intervention coordinator.<br><br>Depression referrals received in each facility were monitored and collected for record keeping on a weekly basis during group supervision. The referral form from the nurse/ doctor to the lay counsellor was used as a source document for this indicator.<br><br>Counselling intervention uptake was recorded on a monthly basis during group supervision sessions. Attendance registers, weekly statistics forms and referral forms were used as source documents.<br><br>In order to help with trouble shooting for the intervention in the Dr KK facilities, in each facility, a committee comprising

	the facility manager, APC trainer, patient who had received three or more sessions, lay counsellor and the intervention supervisor/ mentor was established. This committee met on a monthly basis and discussed the progress of the counselling intervention within the collaborative model of care; the successes and challenges, and came up with resolutions and targets that were followed up during the next meeting. Referral numbers generally picked up in facilities following these meetings. The troubling shooting process was standardized in Bojanala with the use of a common tool.
