# Supplementary material for: A collaborative care package for depression comorbid with chronic physical conditions in South Africa
Source: BMC Health Serv Res. 2022 Dec 1;22:1465. doi: 10.1186/s12913-022-08874-7 (PMC9717432; doi:10.1186/s12913-022-08874-7)
Supplement: Supplementary file 4 — Additional file 4. [file 12913_2022_8874_MOESM4_ESM.pdf]

Supplement 8: TIDieR Template: Orientation of district and primary health care (PHC) facilities to the collaborative care model

**1. Brief name**	**Orientation of facilities and sub-district to the collaborative care model**
**2. Why**	The goal of this orientation was to:<br>• Orientate district and PHC facility managers to the PRIME/CoBALT collaborative model of care<br>• Orientate district and PHC facility to the task-shared intervention<br>• Discuss with facility managers how integration of mental health care could best be achieved in their facilities<br>• Clarify referral pathways<br>• Generate buy-in<br>• Encourage district managers to support facility managers to implement the collaborative care model<br>• Monitor implementation of the collaborative care model (only in Bojanala)
**3. What – material**	1. ***Orientation guide***<br>The orientation was guided by a programme, detailing activities, training goals, step by step facilitation guide, learning objectives, subject areas, methods and trainers.<br>2. ***National Mental Health Policy Framework and Strategic Plan- 2013-2020 (Department of Health, 2013)***<br>3. ***District Mental Health Care Plan (only for Dr KK)(Petersen et al., 2016)***<br>4. ***Results from pilot study in Dr KK (Petersen et al., 2019)***<br>5. ***Video of service user testimony (Ma Agnes) (see*** https://www.youtube.com/watch?v=i79NjZQJ9O8&t=81s***)***<br>6. ***PRIME/CobALT collaborative care model (see Figure 1 in main text) (Dr KK)/PRIME/CobALT infographic (Bojanala)***<br>7. ***APC and Supplementary Mental Health APC (Supplements 1 and 2)***<br>8. ***Clinical Communication Skills Training Manual (Supplement 3)***<br>9. ***Counselling Manual for Depression: The lay-counsellor's guide to depression (Supplement 5)***<br>10. ***Facility monitoring form***

**4. What – procedures**	Orientation half-day workshop used powerpoint presentations, videos as well as discussion (Dr KK and Bojanala). On-site supportive visits (Bojanala only).
**5a. Who provided?**<br><br>**5b. Who received?**	Workshop was facilitated by PRIME/CobALT project staff<br><br>• Intervention facilities managers<br>• District and sub-district managers overseeing intervention facilities
**6. How**	Structured half-day group workshop (Dr KK and Bojanala)<br>On-site supportive visits by project staff (Bojanala only)
**7. Where**	District office
**8. When and how much**	Training was organised to take place before counsellors were placed in their designated facilities to enable facility managers to integrate them into the facility well.
**9. Tailoring**	None
**10. How well – planned**	The orientation workshop was planned to include all intervention facility operational manages, sub-district managers and district managers.
**12. How well – actual**	Dr KK<br>District, sub-district managers and facility managers were well represented at the orientation workshop.<br><br>Bojanala<br>In Bojanala, the workshop was attended by district managers only. Facility managers were engaged individually through on-site visits conducted by study staff prior to any training activities and introduction of the counsellors into the facilities. These visits orientated the facility staff to the intervention components as well as attempting to generate buy-in.<br>The intervention coordinator then supported each facility in the coordination and delivery of the different components on-site, forming the link between Department of Health particularly the Regional Training Centre (Departmental body responsible for in-service training) and the study staff.
**13. Modification**	In Bojanala, the orientation workshop was supplemented with on-site engagement visits with each facility by CobALT staff as well as ongoing support visits that also served to monitor implementation of the collaborative care model.

References

Department of Health (2013). National Mental Health Policy Framework and Strategic Plan 2013-2020. Pretoria, Department of Health, Republic of South Africa.

Petersen, I., Fairall, L., Bhana, A., Kathree, T., Selohilwe, O., Brooke-Sumner, C., . . . Patel, V. (2016). Integrating mental health into chronic care in South Africa: the development of a district mental healthcare plan. Br J Psychiatry, 208 Suppl 56, s29-39. doi: 10.1192/bjp.bp.114.153726

Petersen I, Bhana A, Fairall LR, Selohilwe O, Kathree T, Baron EC, et al. Evaluation of a collaborative care model for integrated primary care of common mental disorders comorbid with chronic conditions in South Africa. BMC Psychiatry. 2019;19(1):107.
